# PyConSolv: A Python Package for Conformer Generation of (Metal-Containing) Systems in Explicit Solvent

**DOI:** 10.1021/acs.jcim.3c00798

**Published:** 2023-08-22

**Authors:** R. A. Talmazan, M. Podewitz

**Affiliations:** † Institute of Materials Chemistry, TU Wien, Getreidemarkt 9, A-1060 Wien, Austria

## Abstract

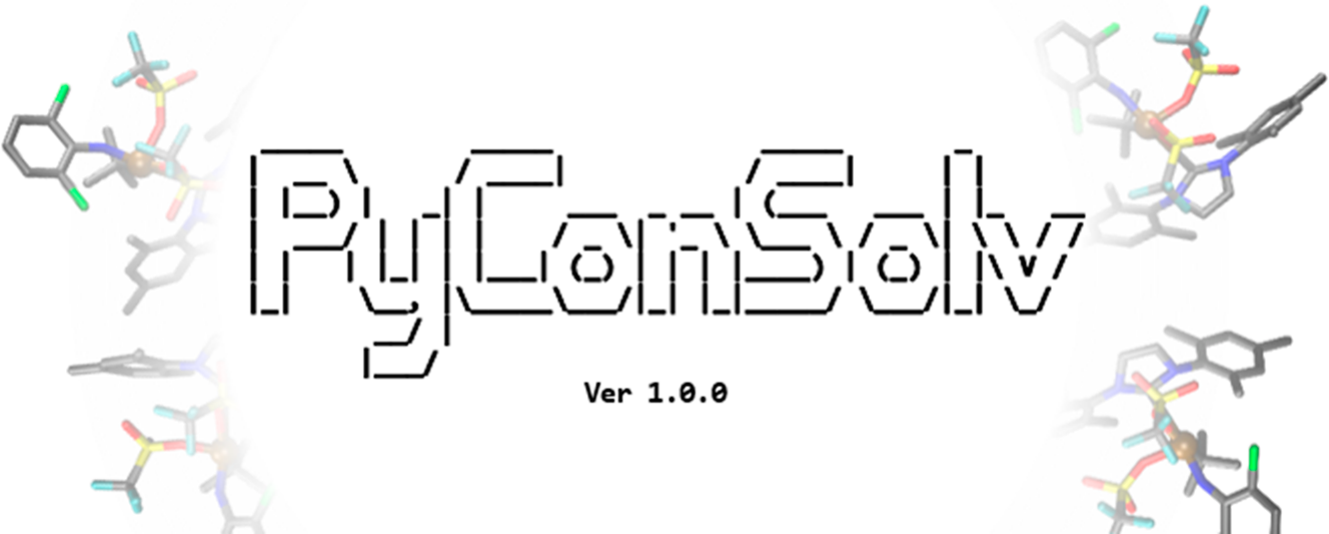

We introduce PyConSolv, a freely available Python package that automates the generation of conformers of metal- and nonmetal-containing complexes in explicit solvent, through classical molecular dynamics simulations. Using a streamlined workflow and interfacing with widely used computational chemistry software, PyConSolv is an all-in-one tool for the generation of conformers in any solvent. Input requirements are minimal; only the geometry of the structure and the desired solvent in xyz (XMOL) format are needed. The package can also account for charged systems, by including arbitrary counterions in the simulation. A bonded model parametrization is performed automatically, utilizing AmberTools, ORCA, and Multiwfn software packages. PyConSolv provides a selection of preparametrized solvents and counterions for use in classical molecular dynamics simulations. We show the applicability of our package on a number of (transition-metal-containing) systems. The software is provided open source and free of charge.

## Introduction

As quantum mechanical (QM) methods and computer hardware evolve and become more efficient, bigger systems,^1−3^ that were previously far out of reach, can be tackled using quantum chemical wave function methods.^4,5^ As the system size increases, it becomes necessary to consider greater flexibility, which in turn presents new challenges that need to be properly addressed.^6,7^ One common approach is to generate conformational ensembles, which can be created in various ways.^8^

While the importance of conformers is well-known and a key feature in drug design pipelines,^9,10^ it has gathered some attention in the field of homogeneous catalysis as early as the 1990s.^11−18^ However, conformer searches are by no means widespread in standard mechanistic studies today. A systematic exploration of the conformational space, by generating rotamers, can be employed for simple systems, with few degrees of freedom, but quickly becomes unfeasible for larger molecules. While several more advanced methods for the generation of conformers exist, they are generally geared toward biological compounds.^19−22^ Methods such as torsional Monte Carlo, augmented with Low Mode searching, offer a way to sample the conformational space, but are limited by computational costs when explicit solvent is necessary.^23,24^ Alternatively, molecular dynamics (MD) methods can be employed for the generation of conformers, by running simulations of the system (in explicit solvent) and clustering the resulting trajectory.^25^ For this purpose, ab initio MD (AIMD)^26^ or classical MD (cMD), in various flavors, can be used, each with its own advantages and disadvantages, as outlined below.

For AIMD, the calculation of the electronic structure is generally considered to be the limiting factor for the overall sampling. To overcome this issue, the use of very efficient semiempirical methods, such as GFN2-xTB,^27^ provides a good compromise. One tailor-made approach for conformer generation is realized in the Conformer Rotamer Ensemble Tool (CREST),^28^ which is shown to perform very well for a large a number of systems,^29^ yet it is not without its downsides. Due to the inherent limitations of semiempirical quantum chemical methods, GFN2-xTB and CREST do not perform well for typical transition-metal systems, and neither structures nor energies are reliable without any further processing.^30^ Another caveat for CREST is the lack of explicit solvation. While implicit solvation models perform admirably for many complexes,^31,32^ they fail to provide reasonable structures for systems containing a cavity,^15^ as intramolecular bonds are heavily favored. This can be counteracted by using explicit solvation, either in microsolvation approaches,^33−35^ that use a few solvent molecules, or in condensed phase calculations, but in any case, at the cost of massively increasing the computational requirements.

On the other hand, cMD simulations rely on a force-field approach, which leads to a great speedup in calculations. Consequently, it allows for explicit treatment of solvent molecules, of course, at some cost in accuracy. While many force fields exist, developed with either specific^36−40^ or general use,^41−45^ running a cMD simulation for metal-containing systems requires the generation of custom parameters for the system under study in order to obtain reasonable results. While there are tools available for the parametrization of organic molecules,^46^ the parametrization of transition metal-containing complexes is a more involved process, requiring a special approach.^47−52^ As metals can form very complex structures, with widely varying coordination numbers,^53^ there are no predefined atom parameters available in the commonly used general force fields. A full parametrization of the metal center is therefore required. One method available in the AmberTools^54^ suite, developed for parametrization of metal-containing biological systems, is the Metal Center Parameter Builder (MCPB).^55^ MCPB utilized a bonded model^47^ to describe the metal ion and its surrounding environment. This approach requires defining the bonded and nonbonded parameters of the force field, for the metal atom and its ligands. These parameters entail structural information, force constants, and potentials for bonds, angles, and dihedrals, as well as partial charges and van der Waals parameters. They are generated based on either experimental or QM optimized structures, by deriving their values from force constants and partial charges.^55^ We would like to stress here that the force-field parameters are generated in such a way that the QM optimized structure is retained. While the use of a bonded model, as implemented in MCPB, provides accurate results for many different (transition-)metal-containing complexes,^14,15,47,55−57^ the generation of parameters is a tedious and error prone process, due to the amount of user intervention that is required.

In this work, we present a user-friendly Python package, which builds upon AmberTools,^54^ to provide an automated process for generating conformers of arbitrary, metal-containing, or metal-free complexes, in explicit solvent. The user only needs to provide an input structure, the desired QM method to be used for the geometry optimization and force constant calculations, the total system charge and multiplicity, and the solvent to be used to the simulation (if applicable). We provide full support for a large number of preparametrized solvents, as well as the ability to parametrize any solvent of choice. Additionally, as many metal-containing complexes are associated with a counterion, we provide seven preparametrized ions, as well as the ability to parametrize an ion of choice. By interfacing to ORCA 5,^58^ the user has access to state-of-the-art QM methods for structure calculations. For system charge assignment, an interface to Multiwfn 3.8,^59^ offers the user a plethora of charge calculations schemes, in addition to the Merz–Kollman RESP scheme^60,61^ recommended by MCPB. After the parametrization is complete, the system is solvated and a suggested equilibration procedure is offered to the user, using the Amber MD package.^62,63^ Once a simulation is performed, the resulting trajectory file can be analyzed via a script provided by PyConSolv. The clustering itself is based on the cpptraj package^64^ and returns a set of conformers, ranked by their QM energy, based on single-point calculations.

## Methodology

In this work, conformational sampling is performed for a number of structures in explicit solvent using PyConSolv to show the applicability as a proof-of-concept method. The systems of choice are a copper containing calix[8]arene catalyst for C–N coupling,^15^ a molybdenum-based catalyst for olefin metathesis,^74,73^ and a metal-free hydrogenobyric acid,^77^ as well as the vitamin B_12_ metabolite methylcobalamin.^78^

### Parametrization

**Figure 1:**
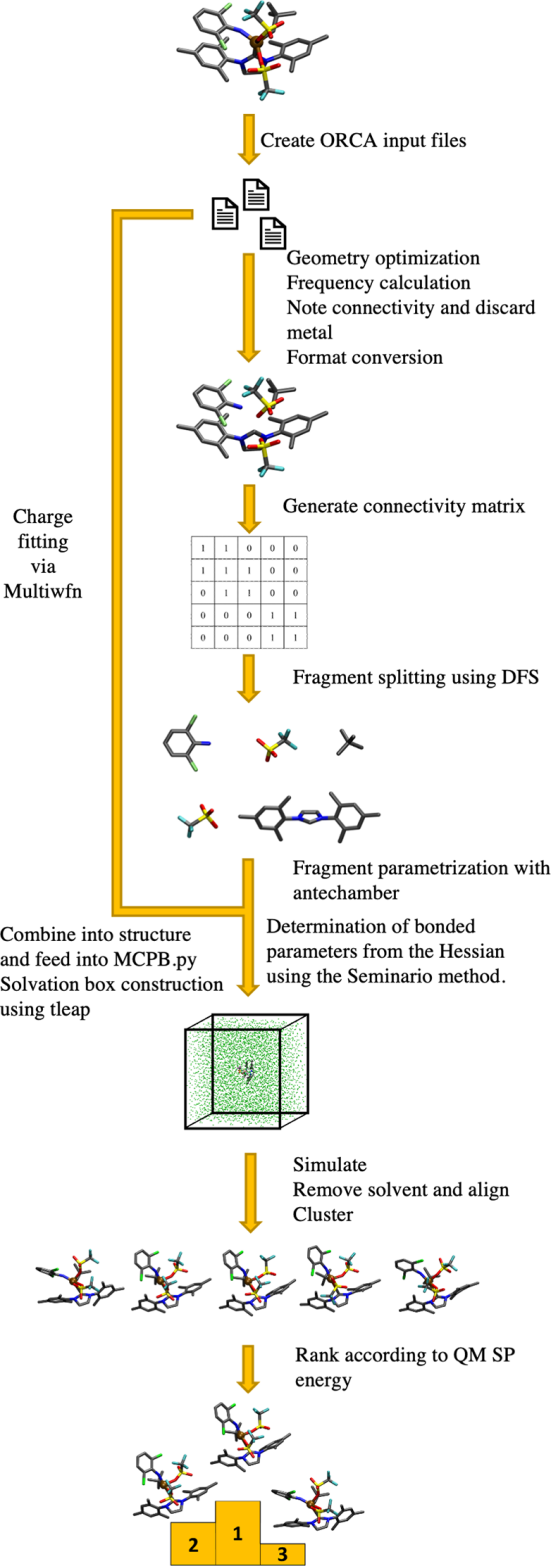
Automated PyConSolv workflow: Generation of the force-field parameters, setup of the simulation box, equilibration, production run, and analysis (for details, see manual in SI).

The protocol implemented in the PyConSolv Python package is shown in Figure 1. The input required is a simple XMOL xyz-formatted molecular structure. Input files for ORCA 5 are generated automatically, and geometry optimization and subsequent frequency calculations are performed at the electronic structure theory level chosen by the user. The optimized geometry is taken as input for subsequent parametrization steps. As the metal center parametrization builds upon the MCPB.py package provided in AmberTools, the steps follow those recommended in the aforementioned package, deriving the force-field parameters using the well-established Seminario method.^65^ The structure must be split into fragments which are to be parametrized separately, with each atom requiring a unique identifier in the pdb files. This step is automated by building a connectivity matrix based on atom radii and pair distances of the atoms. If two atoms are closer together than 60% of the sum of their atomic radii, they are considered to be bonded. If one of the atoms is a metal, the connectivity is checked, but it is not added to the matrix, as the metal needs to be parametrized separately from the rest of the fragments. Using a depth first search algorithm^66^ to traverse the connectivity matrix, we are able to identify each individual fragment. The user is then presented with an interactive window, where a Lewis structure of each fragment is displayed. Here, we require the user to provide total fragment charges to perform the parametrization of each individual fragment using antechamber.^46^ The ORCA 5 output files are analyzed and converted into inputs for Multiwfn 3.8 and MCPB, also accounting for the usage of effective core potentials^67^ for heavy atoms. The RESP charges for the system are calculated using Multiwfn, with the recommended settings, as in the Multiwfn user manual (see SI). The MCPB input file is created, taking into account the carbon–metal bonds present in the system, which are not natively recognized. The metal center and all atoms directly bound to it have a new atom type assigned. The bonded parameters are derived from the Hessian matrix of the system using the Seminario method.^65^ The previously calculated RESP charges are taken into account and enter the nonbonded part of the force-field parameters. For the ligands, the initial guesses for the parameters are based on modifications of the GAFF2 force-field parameters. Using the parameters generated by MCPB, a simulation box is then set up, with either one of the 18 preconfigured solvents or any other user-defined solvent. Subsequently, an equilibration script, which follows an extensive heating and cooling procedure as outlined by Wallnoefer et al.,^68^ is provided, as well as an input file for a simulation of 100 ns in the NVT ensemble, with *T* = 300 K.

For a detailed guided workflow, please refer to the documentation in the SI or the online information available on GitHub (https://github.com/PodewitzLab/PyConSolv).

### Supported Solvents and Ions

As part of the PyConSolv package, the solvents listed in Table S1 (SI) have been preparametrized and it has been verified that the equilibrated solvent box densities at 300 K are close to the experimentally determined densities. All parametrizations were performed on structures optimized with BP86/def2-SVP/D4,^69−72^ with the exception of acetonitrile, for which no D4 corrections were used, as the resulting structure was incorrect. For parametrization, antechamber was used, with RESP charges.

In addition to solvents, the following common counterions have been parametrized: ScF_6_^3–^, BF_4_^–^, B[Ar^F^]_4_^–^, B(Ph)_4_^–^, PF_6_^–^, OTf^–^, and ClO_4_^–^. The structures were optimized with BP86/def2-SVP/D4^69−72^ and all charges were computed with RESP. During parametrization for BF_4_^–^ and ClO_4_^–^, the angle parameters could not be determined automatically with antechamber/MCPB.py. In these cases, a scan of the angle was performed with ORCA and the parameter were assigned manually.

## Analysis

After a cMD production run is completed, the resulting trajectory can be analyzed using PyConSolv. This can be done in two ways, either using one of the provided shell scripts or using the Python implementation present within PyConSolv. To obtain relevant conformers, the trajectory must be clustered. As we use a root-mean-square deviation (RMSD) of the distance of all heavy atoms as the clustering criteria, it is vital that the trajectory is properly aligned. This is resolved by aligning the complex based on a list of atom indices, provided by the user. The clustering can then be performed using one of the four methods provided by cpptraj: dbscan, hierarchical, k-means, or dpeaks.^64^ On the resulting clusters, single-point calculations are performed, using the same electronic structure theory level that was used during the parametrization, including implicit solvation and dispersion corrections, as detailed in each test case. Finally, a list of clusters, ranked by the single-point energy, is presented to the user.

## Results

The PyConSolv workflow, as explained above, was applied to the following systems: a Cu(I)-calix[8]arene,^15^ a Mo imido alkylidene N-heterocyclic carbene catalyst,^73,74^ hydrogenobyric acid,^75−77^ and methylcobalamin.^78^ A suitable functional was chosen for each system, along with the appropriate solvent, as to mimic the experimental conditions. All charges were calculated using RESP. After parametrization, the system was equilibrated, and a 100 ns cMD production run was performed, using the simulation input file provided by PyConSolv. The clusters were generated using k-means, with the script provided by PyConSolv, using the RMSD of all nonhydrogen atoms as the distance metric. The simulations were aligned on the “rigid” parts of the structure, as described in each case below. A total of 10 clusters were chosen for each system and evaluated.

As a note about runtime, when comparing PyConSolv with CREST, it can be seen that with increase in system size, the PyConSolv workflow becomes noticeably faster, as the time-consuming step becomes the frequency calculation, rather than the cMD simulation (see SI Table S2 for timings).

### Case 1: Cu(I)-Calix[8]arene

Cu(I)-phenanthroyl encapsulated by calix[8]arene is a catalyst that has proven to be very complex to model, with the macrocyclic cage being particularly mobile. This inherent flexibility needs to be taken into account, in order to obtain an accurate energy profile for the reaction^15^ or to explain the difference in activity between various regioisomers.^14^

**Figure 2:**
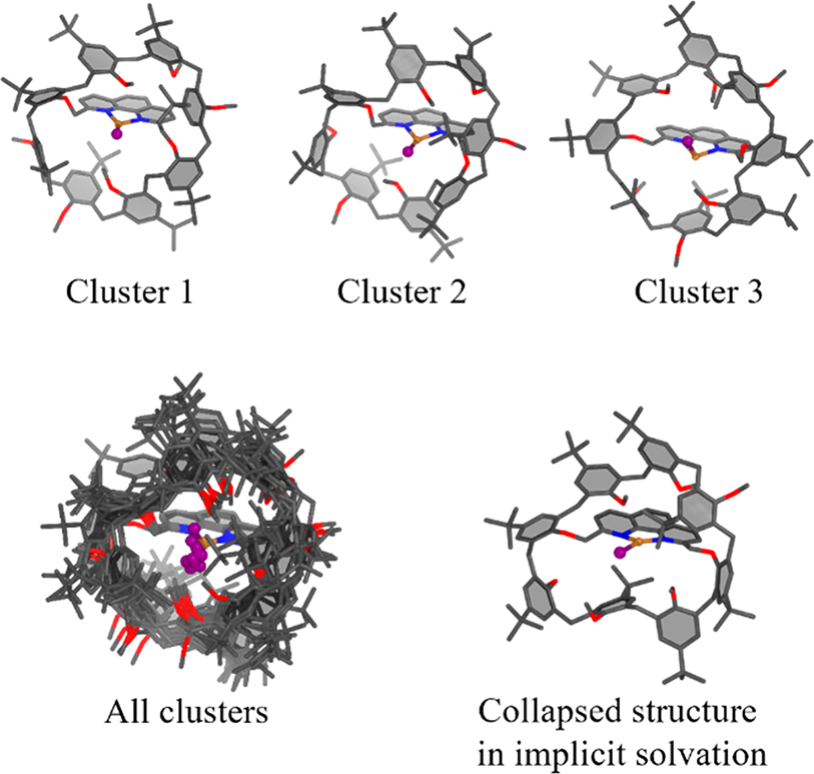
Cu(I)-calix[8]arene clusters. Upper panel: Top three clusters with lowest QM energy. Lower panel: All 10 clusters superimposed (left). The bottom right image depicts a collapsed cage structure in implicit solvation.

Using PyConSolv, with PBE0/def2-SVP/D3+CPCM(Chloroform),^31,72,79,80^ the system shown in Figure 2 was parametrized and solvated in chloroform. The trajectory was aligned on the copper and phenanthroline moieties. The clusters shown in Figure 2 represent those with the lowest energy, as computed via DFT single points, with the above specified density functional, basis set, and dispersion correction. For comparison, structures generated with CREST using GFN2-xTB and implicit solvation (chloroform) have been generated as well. From the representative cluster depicted in Figure 2, it can be seen that the calix[8]arene cage is collapsed around the copper center, when compared to the structures generated with PyConSolv. This is an artifact caused by the lack of explicit solvation in the CREST conformers and the tendency of dispersion corrections to favor compact structures with many intramolecular interactions. The collapsed structure is in stark contrast to those generated in explicit solvent via PyConSolv (see Figure 2, bottom left). As it is known from experiments that an intact cavity is vital for any catalysis to take place,^14,15^ PyConSolv yields structures that are a better representation of the solution phase conformations.

### Case 2: Molybdenum Imido Alkylidene N-Heterocyclic Carbene (NHC) Catalyst

Molybdenum imido alkylidene N-heterocyclic carbene (NHC) catalysts represent one avenue of performing olefin metathesis.^73,74^ It was shown during the investigation of the reaction mechanism, that conformer generation is crucial for explaining the reactivity.^16^

**Figure 3:**
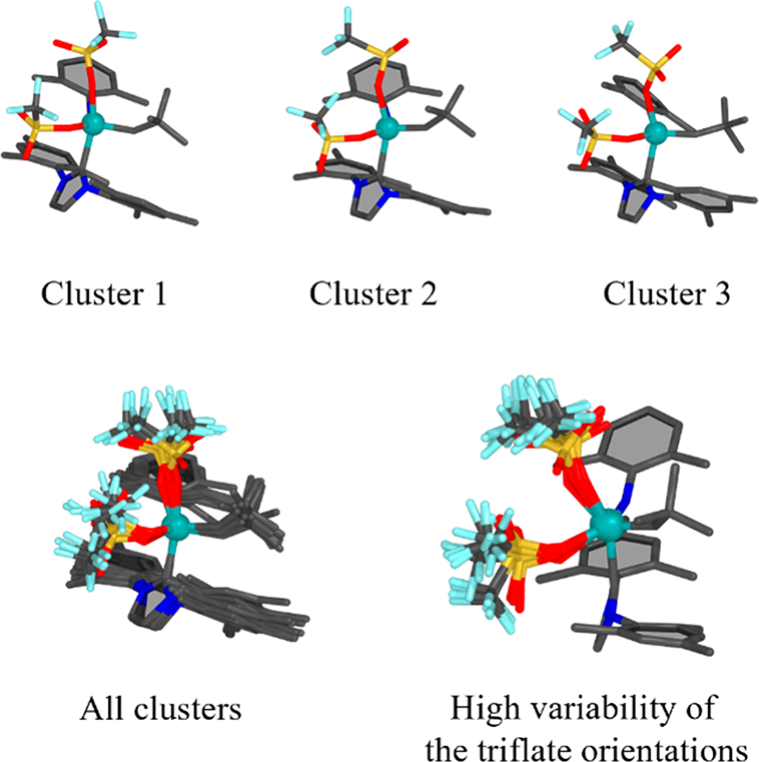
Mo imido alkylidene NHC catalyst clusters. Upper panel: Top three ranked clusters ranked by QM energy. Lower panel: All 10 clusters superimposed (left). The bottom right image highlights the high flexibility of the triflate groups.

For the Mo-based catalyst, the structures generated with PyConSolv used BP86/def2-SVP/D3BJ+CPCM(CH_2_Cl_2_)^31,69,70,72,81^ for the force-field parametrization. The trajectory was aligned on the molybdenum, the carbon of the aryl group, and the NHC carbon bound to the molybdenum. It can be seen that the simulation captured the movement of the ligands, as shown in Figure 3. The triflate ligands show the most prominent flexibility. For comparison, structures were generated with CREST as well (see Figure S1, Supporting Information). The movement of the triflate groups is more pronounced in the CREST conformers, likely due to the enhanced sampling method implemented within its code. While similar enhanced sampling methods can easily be utilized in conjunction with PyConSolv, this is beyond the scope of this work. The NHC moiety, along with the aryl ligand, appear to be considerably more rigid. While the CREST conformers display a rotation in the NHC group, this is not present in the cMD simulation set up by PyConSolv. However, the underlying electronic structure method of CREST, GFN2-xTB, has shown to be less appropriate for this particular catalyst, and as such, the resulting structures require further refinement using DFT. In constrast, the Mo center coordination geometry in the force field is constrained to QM optimized one. Consequently, an adequate structure is conserved in the MD simulations at the cost that changes in the Mo coordination geometry cannot be captured. Yet, both sets of conformers look rather similar at first glance; hence, the solvent does not seem to have a great influence on the overall structure of the catalyst.

### Case 3: Hydrogenobyric Acid

Hydrogenobyric acid represents the metal-free precursor of vitamin B_12_ from which numerous native or artificial metal-cobalamins can be synthesized.^75−77^ Its structural variability may prove to be of interest for the design of new drugs.^82,83^ The electronic structure calculation for parametrization was performed with BP86/def2-SVP/D4+CPCM(Water)^31,69−72^ and the trajectory was aligned on the nitrogen atoms of the corrin ring.

**Figure 4:**
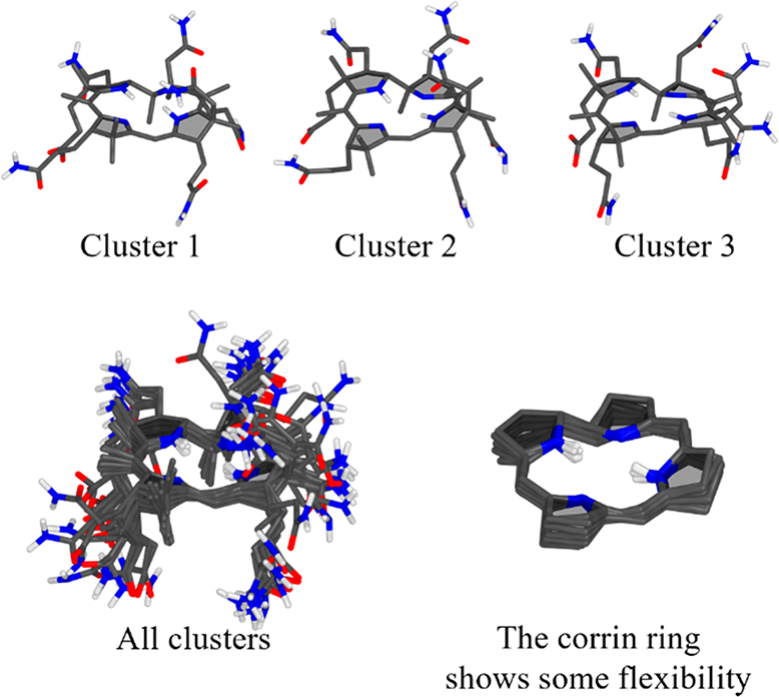
Hydrogenobyric acid clusters. Upper panel: Top three clusters with lowest QM energy. Lower panel: All 10 clusters superimposed (left) and a view of the corrin ring of the clusters (right).

It can be seen from Figure 4 that the flexibility of the side chains is quite well captured, along with the flexibility of the corrin ring. The test case also illustrates that PyConSolv works equally well for metal-free systems.

### Case 4: Methylcobalamin

Methylcobalamin is a vitamin B_12_ metabolite.^78^ For the PyConSolv procedure, as in case 3, for parametrization, BP86/def2-SVP/D4+CPCM(Water)^31,69−72^ was the method of choice. The alignment was performed, as before, on the nitrogen atoms of the corrin ring.

**Figure 5:**
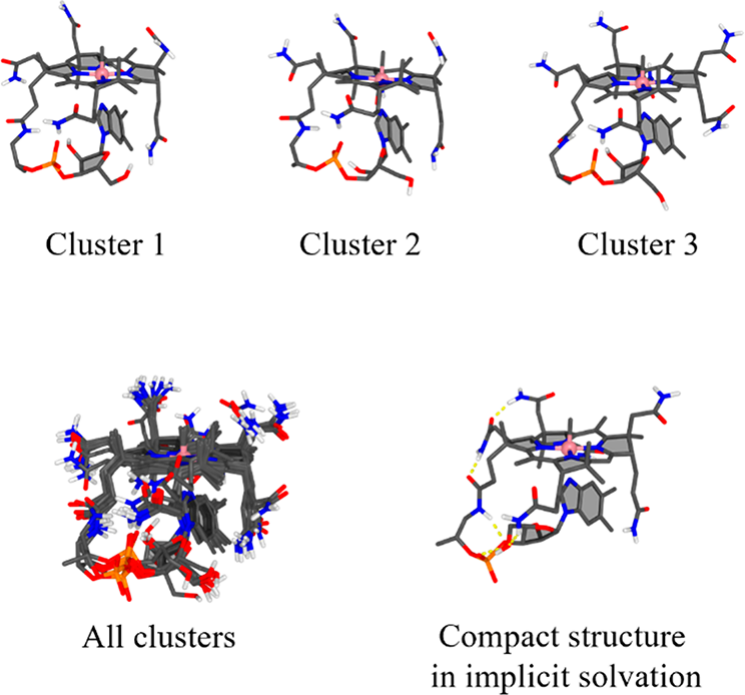
Methylcobalamin clusters. Upper panel: Top three clusters ranked by QM energy. Lower panel: All 10 clusters superimposed (left) and an example of a structure optimized in implicit solvent, dominated by intramolecular hydrogen bonding (right).

It can be seen from the clusters in Figure 5 that the methylcobalamin structure is fairly rigid. The stabilization of the corrin ring is apparent when compared to that of the hydrogenobyric acid. It can be seen that all amide groups show some flexibility, while always pointing toward the solvent.

For sake of comparison, investigation of the complex in implicit solvent yielded a structure with many intramolecular hydrogen bonds of the amides and a loop conformation that is very different to the ones in explicit solvent (see Figure 5 bottom right; Figure S2, Supporting Information). These intramolecular hydrogen bonds may affect the reactivity of the complex, and it is unlikely that they are formed under experimental conditions. Consequently, PyConSolv provides more realistic liquid phase structures here.

### Conformer Evaluation

The conformations generated by clustering of the trajectory are subjected to single-point energy calculations at the same level of theory as the parametrization to determine a ranking. Although this is a simplistic approach, it provides a good first guess as to which structures should be used for further refinement. While this is a fast method, it ignores the effects that explicit solvation might have on the overall stability of the system. To account for this, one would have to move to more complicated and costly methods such as QM/MM embedding schemes or resort to microsolvation models. An interface to the FEBISS^34^ microsolvation package is planned for the near future.

## Conclusion

In this work, we present a Python package to automate conformer generation of (metal-containing) complexes in explicit solvent. Our tool performs parametrization of metal-containing structures and sets up a workflow for simulation and analysis. The automatization makes conformer generation accessible to the nonexpert user. It allows for a straightforward implementation of conformational sampling (in explicit solvent) in standard computational chemistry workflows, e.g., for the determination of reaction mechanisms. With increasing system size, our Python package outperforms semiempirical methods, such as CREST. A subsequent reoptimization of the obtained clusters with DFT ensures that true energy minima are found.

While standard classical MD simulations can sometimes have difficulties in exhaustively exploring the potential energy surface, especially when the structure is trapped in a deep minimum, this obstacle can be overcome by resorting to enhanced sampling methods such as accelerated molecular dynamics^84^ or metadynamics.^85^ Alternatively, a more complex approach of Monte Carlo with Low Mode search, can be combined with MD to speed up the PES exploration. These methods add considerable complexity and are not currently implemented in PyConSolv. Another extension would be the implementation of MM methods, which would allow for the changes of the coordination around the metal center,^86,87^ to switch between different coordination geometries to better reflect the nature of the metal atoms.

Nevertheless, the presented case studies show that conformer generation in explicit solvent leads to more realistic representations of the liquid phase structure. Our tool is a stepping stone toward more realistic modeling of reaction mechanisms and thus a prerequisite for calculations to become predictive.

## Data Availability

The PyConSolv package is available open source, free of charge, on GitHub: https://github.com/PodewitzLab/PyConSolv. Likewise, it can be installed from the popular PyPi software repository using pip install PyConSolv.

## Supporting Information Available

Details of computational methodology including settings used for Multwfn, CREST, and solvent parametrization, as well as for the equilibration of MD simulations with AMBER; additional comparison between implicit and explicit solvent conformers; tutorial on how to use PyConSolv (PDF)
