# Supplementary material for: PyConSolv: A Python Package for Conformer Generation of (Metal-Containing) Systems in Explicit Solvent
Source: J Chem Inf Model. 2023 Aug 22;63(17):5400–7. doi: 10.1021/acs.jcim.3c00798 (PMC10498442; doi:10.1021/acs.jcim.3c00798)
Supplement: Supplementary file 1 — ci3c00798_si_001.pdf [file ci3c00798_si_001.pdf]

Supporting Information:

PyConSolv:

A Python Package for Conformer Generation of (Metal-Containing) Systems in Explicit Solvent

R. A. Talmazan[1] *and M. Podewitz**[1]

[1] Institute of Materials Chemistry, TU Wien, Getreidemarkt 9, A-1060 Wien (Austria), maren.podewitz@tuwien.ac.at

Multiwfn charge settings

Multiwfn 3.8[1] is used to compute the charges for the parametrizations, as follows.

Nuclear + Electronic Merz-Kollman RESP[1] type atomic charge mode is used with 6 points/Å^2. All atoms are used for fitting 4 layers, with the scaling factors: 1.4,1.6,1.8,2.0. Automatic radii are utilized for fitting, missing radii are taken from UFF and scaled by 1/1.2. For fitting, the tightness parameter is 0.1, the restraint strengths for the two stages are set to 0.0005 and 0.0010, respectively. The maximum number of iterations is 50 and the convergence threshold is set to 10^{-6}. Charge equivalence constraints are enabled for CH_2 and CH_3 groups. No charge constraint settings are used and the connectivity is determined automatically.

CREST settings

CREST[2] was used for generating conformations as comparisons for the chosen test cases. For this, the GFN2-xTB[3] method was chosen, using ALPB[4] as the solvent model, with an appropriate solvent for each system. The energy threshold, RMSD and conformer pair energy differences were set to 6 kcal/mol, 0.125Å and 0.05 kcal/mol, as detailed in the CREST documentation. For the case of the Mo-catalyst, the energy threshold was raised to 50 kcal/mol, because a low number of conformers was obtained.

Solvent Parametrization

The solvents were parametrized using RESP charges and antechamber, after which they were equilibrated and a short, 10ns, classical molecular dynamics (cMD) run was performed to examine the density.

<u>Counterions.</u> The counterions shown in Figure S1 ($B[Ar^F]_4^-$, BF_4^-, OTf^-, ScF_6^{3-}, ClO_4^-, PF_6^-, and $B(Ph)_4^-$) have been parametrized and are available in PyConSolv.

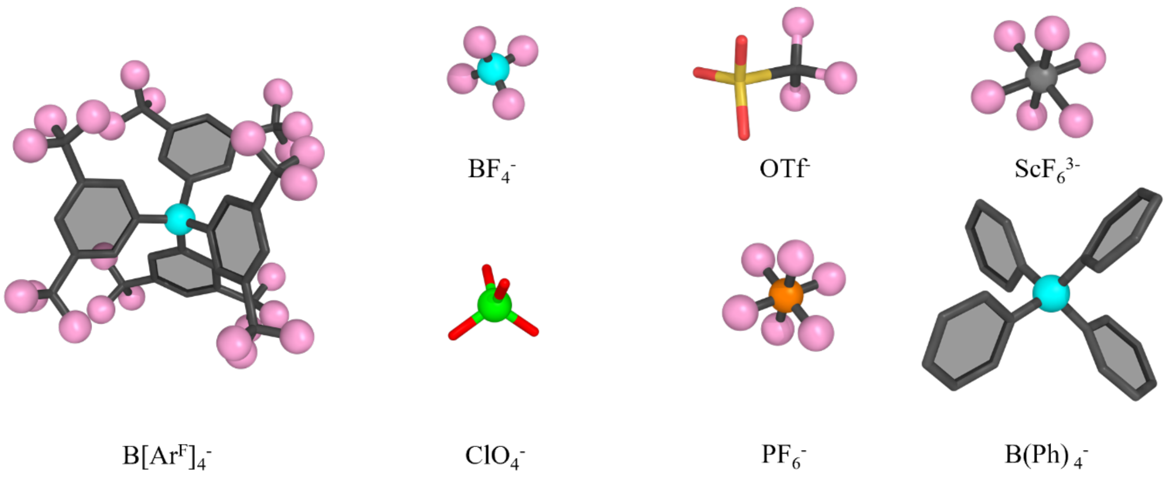

Figure S1 Structures of parametrized counterions; color coding: C atoms in grey, B atoms in cyan, F in pink, S in yellow, O in red, Sc in silver, P in orange.

Table S1. Density of boxes with parametrized solvent, after 10ns cMD. Densities taken from CRC Handbook of Chemistry and Physics, 95th Edition.[5] * denotes gas phase, while the calculated density is in the condensed phase.

Solvent	Exp. Density [g/cm^3]	Calc. Density [g/cm^3]	Density standard deviation [g/cm^3]
Acetone	0.7845	0.8160	0.0016
Acetonitrile	0.7857	0.8261	0.0013
Ammonia	0.696*	0.9523	0.0014
Benzene	0.8765	0.8519	0.0022
Tetrachloromethane	1.5940	1.5659	0.0036
Dichloromethane	1.3266	1.2617	0.0026
Chloroform	1.4788	1.4365	0.0029
Cyclohexane	0.7739	0.7496	0.0024
DMF	0.9445	0.9950	0.0020
DMSO	1.1010	1.1492	0.0022
Ethanol	0.7893	0.8142	0.0017
Hexane	0.6606	0.6339	0.0018
Methanol	0.7914	0.8171	0.0017
1-Octanol	0.8262	0.8180	0.0029
Pyridine	0.9819	0.9937	0.0022
THF	0.8833	0.8912	0.0019
Toluene	0.8623	0.8411	0.0025

Timings

The timing for the complete run of PyConSolv for each the testcase system is detailed in Table S2. The calculations were performed on a Ryzen R9 5900x CPU, using 12 threads and a total system memory of 64 GB. The simulations were performed using the GPU accelerated implementation of pmemd[6] on an RTX 3060 GPU.

Table S2. Timings for electronic structure calculations and simulation steps for PyConSolv and CREST.

System	PyConSolv						CREST
	SCF Cycle / h	Frequency Calculation / h	Antechamber / h	Equilibration / h	Simulation / h	Total / h	Total / h
**Cu(I)-Calix[8]arene**	0.11	17.84	3.08	0.67	9.01	30.71	483.32
**Mo-NHC**	0.05	1.07	0.08	0.33	8.45	9.98	10.68
**Hydrogenobyric Acid**	0.04	0.00	0.08	0.77	14.05	14.95	42.21
**Methylcobalamin**	0.14	5.91	0.50	1.75	22.53	30.83	423.52

Amber MD settings

To perform the classical molecular dynamics simulations, the Amber20[6] package was used, in combination with AmberTools23[6]. For the system equilibration, a multi-step approach with repeated heating and cooling was chosen, as follows:

1. The entire simulation box is restrained using cartesian restraints (restraint weight = 1000 kcal mol^{-1} $Å^{-2}$), with the exception of the hydrogen atoms, which undergo an energy minimization for 500 cycles.
2. The solute heavy atoms remain restrained and the whole system is minimized for 1000 cycles (restraint weight = 1000 kcal mol^{-1} $Å^{-2}$).
3. The system is equilibrated under constant volume to achieve a temperature of 300K, using Langevin dynamics[7] with the collision frequency of 2.0 ps^{-1}, a timestep of 0.001 ps and the SHAKE[8] algorithm for hydrogen bonds.
4. The solute heavy atoms remain restrained (restraint weight = 1000 kcal mol^{-1} $Å^{-2}$) and the whole system is equilibrated at constant pressure (1 atm), to achieve a reasonable system density and eliminate the voids created by the automated solvent placement in tleap. This is achieved using Langevin dynamics with the collision frequency of 2.0 ps^{-1}, a timestep of 0.001 ps and the SHAKE algorithm for hydrogen bonds.
5. The system is subsequently cooled under constant volume to achieve a temperature of 100K, using Langevin dynamics with the collision frequency of 2.0 ps^{-1}, a timestep of 0.001 ps and the SHAKE algorithm for hydrogen bonds.
6. A stepwise reduction of the restraints takes place, from 1000 to 0 kcal mol^{-1} $Å^{-2}$, minimizing at each step (1000, 500, 200, 100, 50, 20, 10, 5, 4, 3, 2, 1, 0.5 kcal mol^{-1} $Å^{-2}$)
7. The system is heated up under constant volume to achieve a temperature of 300K, using Langevin dynamics with the collision frequency of 2.0 ps^{-1}, a timestep of 0.001 ps and the SHAKE algorithm for hydrogen bonds.

8. A final constant pressure (1 atm) equilibration takes place, using Langevin dynamics with the collision frequency of 2.0 ps^{-1}, a timestep of 0.001 ps and the SHAKE algorithm for hydrogen bonds.

The simulations then take place under constant pressure (1 atm, Berendsen barostat[9]) with isotropic position scaling, a pressure relaxation time of 2.0 ps with Langevin dynamics, SHAKE for the hydrogen atoms and a timestep of 0.001 ps.

The size of the simulation box is automatically detected based on the size of the system and a cubic shape is chosen in all instances. These settings resulted in the following number of particles for the simulations: Cu(I)-Calix[8]arene in chloroform: 966; molybdenum N-heterocyclic carbene (NHC) catalyst in dichloromethane: 844; hydrogenobyric acid in water: 5529; methylcobalamine in water: 6766.

Case 2: Molybdenum N-heterocyclic carbene (NHC) catalyst

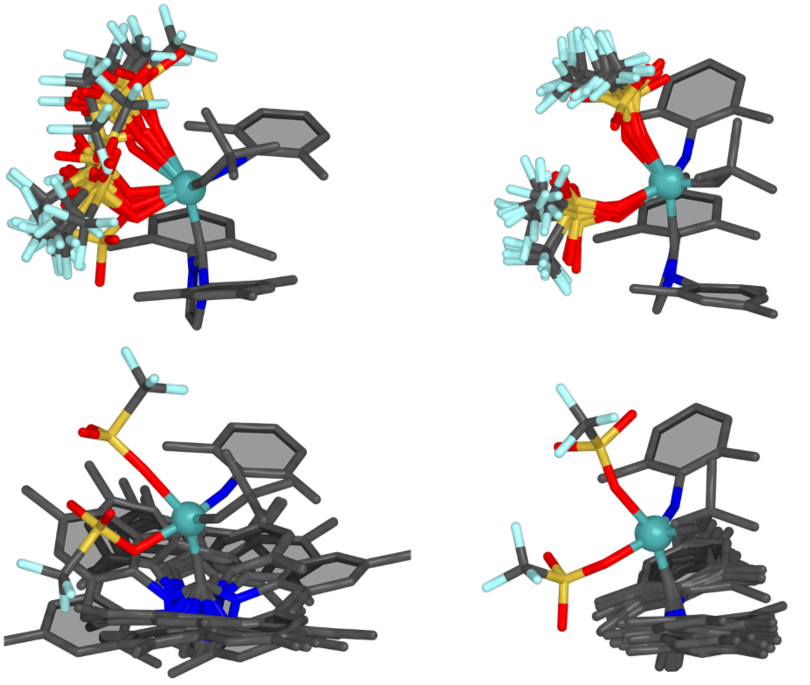

CREST Conformers PyConSolv Conformers

Figure S2 Comparison of conformers generated with CREST and PyConSolv.

In the case of the Molybdenum catalyst, it is apparent that CREST[2] achieves a greater sampling for the conformational space, thanks to its metadynamics based approach. PyConSolv can achieve further sampling through prolonging the classical molecular dynamics simulations or employing enhanced sampling techniques.

Case 4: Methylcobalamin

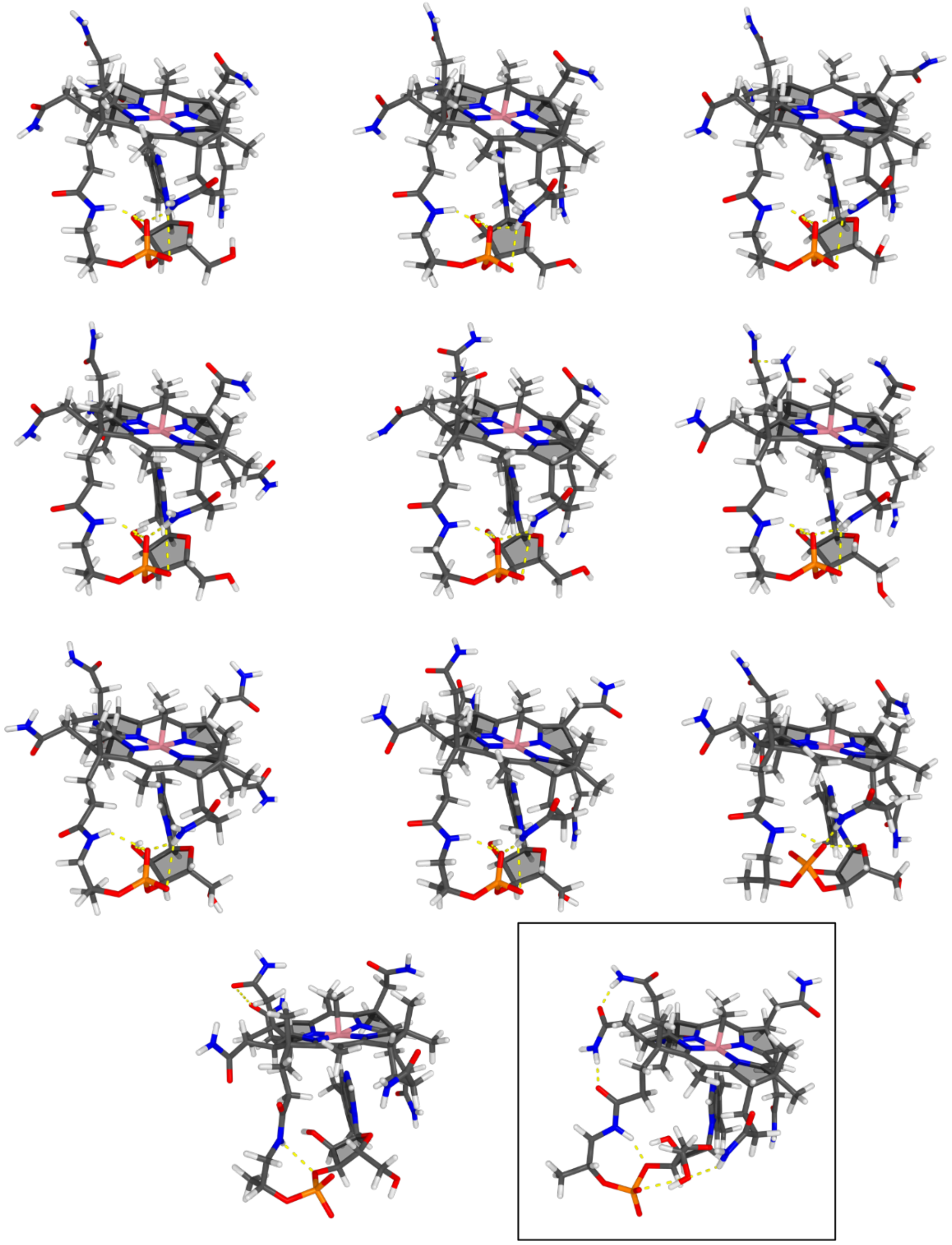

Figure S3. Conformers generated with PyConSolv, compared to a conformer with significant intramolecular hydrogen bonding from implicit solvation DFT optimizations (black rectangle).

Because of to the use of explicit solvation, unphysical intramolecular hydrogen bonding is minimized, when conformers are generated using PyConSolv, as shown in Figure S3.

Package Usage

The package was installed following the instructions on the github page(https://github.com/PodewitzLab/PyConSolv).

A new folder for the chosen system was created and the structure was saved as an XMOL file (input.xyz). This serves as the input for the pyconsolv command.

The parametrization was started using the following command: "pyconsolv input.xyz -m MMM -b BBB -s SSS -d DDD", where MMM represents the DFT functional, BBB represents the basis set, SSS represents the solvent and DDD represents the dispersion correction model.

After the geometry optimization and frequency calculation are complete, a pop-up window appears, where the charges for each of the detected fragments are assigned (see Figure S4).

Following the charge assignment, the parameters are created, the system solvated and the equilibration is automatically started.

The simulation run is started in the newly created simulation folder, using the "run_simulation.sh" script, generated by PyConSolv.

Following the 100ns cMD production run, the analysis was performed using "pyconsolv -a sim-01", choosing kmeans as the clustering method and default values (10 mininum points per cluster, 10 clusters total).

For a more detailed guide, please consult the user manual provided with PyConSolv, which can be found on the github repository.

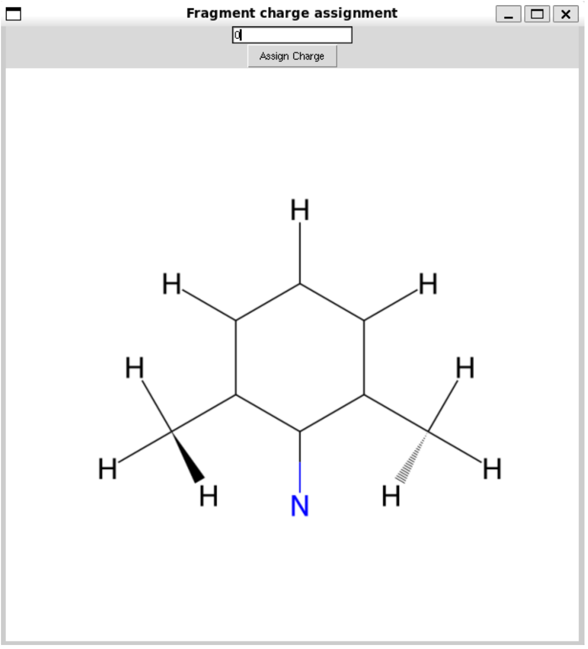

Figure S4 Fragment charge assignment dialogue. Here the user must provide the charges for each fragment of the initial molecule.

References

(1) Lu, T.; Chen, F. Multiwfn: A Multifunctional Wavefunction Analyzer. *J. Comput. Chem.* **2012**, *33* (5), 580–592. https://doi.org/10.1002/jcc.22885.

(2) Pracht, P.; Bohle, F.; Grimme, S. Automated Exploration of the Low-Energy Chemical Space with Fast Quantum Chemical Methods. *Phys. Chem. Chem. Phys.* **2020**, *22* (14), 7169–7192. https://doi.org/10.1039/C9CP06869D.

(3) Bannwarth, C.; Ehlert, S.; Grimme, S. GFN2-xTB—An Accurate and Broadly Parametrized Self-Consistent Tight-Binding Quantum Chemical Method with Multipole Electrostatics and Density-Dependent Dispersion Contributions. *J. Chem. Theory Comput.* **2019**, *15* (3), 1652–1671. https://doi.org/10.1021/acs.jctc.8b01176.

(4) Ehlert, S.; Stahn, M.; Spicher, S.; Grimme, S. Robust and Efficient Implicit Solvation Model for Fast Semiempirical Methods. *J. Chem. Theory Comput.* **2021**, *17* (7), 4250–4261. https://doi.org/10.1021/acs.jctc.1c00471.

(5) Haynes, W. M. *CRC Handbook of Chemistry and Physics, 95th Edition*, 95th ed.; CRC Press: Hoboken, **2014**.

(6) Case, D. A.; Ben-Shalom, I. Y.; Brozell, S. R.; Cerutti, D. S.; Cheatham III, T. E.; Cruzeiro, V. W. D.; Darden, T. A.; Duke, R. E.; Ghoreishi, D.; Gilson, M. K. AMBER20. *University of California, San Francisco* **2020**.

(7) Adelman, S. A.; Doll, J. D. Generalized Langevin Equation Approach for Atom/Solid-surface Scattering: General Formulation for Classical Scattering off Harmonic Solids. *J. Chem. Phys.* **2008**, *64* (6), 2375–2388. https://doi.org/10.1063/1.432526.

(8) Ciccotti, G.; Ryckaert, J. P. Molecular Dynamics Simulation of Rigid Molecules. *Computer Physics Reports* **1986**, *4* (6), 346–392. https://doi.org/10.1016/0167-7977(86)90022-5.

(9) Berendsen, H. J. C.; Postma, J. P. M.; van Gunsteren, W. F.; DiNola, A.; Haak, J. R. Molecular Dynamics with Coupling to an External Bath. *J. Chem. Phys.* **1984**, *81* (8), 3684–3690. https://doi.org/10.1063/1.448118.
